# Is distance associated with tuberculosis treatment outcomes? A retrospective cohort study in Kampala, Uganda

**DOI:** 10.1186/s12879-020-05099-z

**Published:** 2020-06-11

**Authors:** Katherine O. Robsky, Seamus Hughes, Alex Kityamuwesi, Emily A. Kendall, Peter James Kitonsa, David W. Dowdy, Achilles Katamba

**Affiliations:** 1 Department of Epidemiology, Johns Hopkins Bloomberg School of Public Health, Baltimore, USA; 2 Uganda Tuberculosis Implementation Research Consortium, Kampala, Uganda; 3 Johns Hopkins School of Medicine, Baltimore, USA; 4 Clinical Epidemiology and Biostatistics Unit, Department of Medicine, Makerere University, College of Health Sciences, Kampala, Uganda

**Keywords:** Epidemiology, Health systems research, Geographic information systems

## Abstract

**Background:**

Challenges accessing nearby health facilities may be a barrier to initiating and completing tuberculosis (TB) treatment. We aimed to evaluate whether distance from residence to health facility chosen for treatment is associated with TB treatment outcomes.

**Methods:**

We conducted a retrospective cohort study of all patients initiating TB treatment at six health facilities in Kampala from 2014 to 2016. We investigated associations between distance to treating facility and unfavorable TB treatment outcomes (death, loss to follow up, or treatment failure) using multivariable Poisson regression.

**Results:**

Unfavorable treatment outcomes occurred in 20% (339/1691) of TB patients. The adjusted relative risk (aRR) for unfavorable treatment outcomes (compared to treatment success) was 0.87 (95% confidence interval [CI] 0.70, 1.07) for patients living ≥2 km from the facility compared to those living closer. When we separately compared each type of unfavorable treatment outcome to favorable outcomes, those living ≥2 km from the facility had increased risk of death (aRR 1.42 [95%CI 0.99, 2.03]) but decreased risk for loss to follow-up (aRR 0.57 [95%CI 0.41, 0.78]) than those living within 2 km.

**Conclusions:**

Distance from home residence to TB treatment facility is associated with increased risk of death but decreased risk of loss to follow up. Those who seek care further from home may have advanced disease, but once enrolled may be more likely to remain in treatment.

## Background

Although tuberculosis (TB) is both preventable and treatable, it is the leading cause of death due to a single pathogen worldwide [1]. One challenge in TB control is maintaining adherence to a minimum of six months of treatment. The World Health Organization estimates that 82% of patients worldwide who start treatment for TB experience treatment success (defined as treatment completion or cure); this percentage has not improved substantively in the past decade [1]. People with restricted access to health care may not be able to initiate and complete TB treatment as recommended. Risk factors for unfavorable treatment outcomes include demographic, clinical, and health systems characteristics [2–4].

Geographic barriers to care, including physical distance to a health facility, may contribute to poor treatment outcomes. Distance from home to health facility has been associated with decreased access to a wide range of health services and outcomes, including poor HIV treatment clinic attendance [5] and antiretroviral adherence [6], lower likelihood of facility-based childbirth [7], and maternal [8] and child mortality [9]. Geographic barriers to care have been linked to delays [6, 10], loss-to follow up [11], and lack of adherence during the TB diagnostic evaluation and treatment processes [10]. Whether these associations translate into worse treatment outcomes remains uncertain. As the effect of geographic barriers has primarily been noted in rural areas where patients likely have limited options as to where they seek care and even the closest health care facilities may require more than an hour of travel, we aimed to understand the effect of these barriers on TB treatment outcomes in the context of a densely populated, urban setting where there are many TB treatment facilities available and patients may choose to seek care at facilities other than the ones closest to them.

Understanding links between geographic barriers to care and TB treatment outcomes may help identify a population at risk for unfavorable treatment outcomes that could be targeted with interventions to reduce barriers to care and improve outcomes. We therefore sought to characterize the association of distance from home to health facility and TB treatment outcomes in six public and private health facilities in Makindye division, Kampala district, Uganda.

## Methods

### Study overview and population

We conducted a retrospective cohort study of TB patients at one public (Kisugu Health Center, the primary public TB treatment facility in this area) and five private urban outpatient health facilities serving the population of Kisugu and Wabigalo parishes of Makindye division, Kampala, Uganda. Facilities were included if they provided TB care to an average of at least one patient per month from Kisugu or Wabigalo parish. At each of the selected facilities, all patients initiating TB treatment from January 1, 2014 to December 31, 2016 and who lived in Uganda were included. The National TB Control Program oversees all TB care and patients may seek treatment at any facility of their choosing. Treatment of adult TB is largely decentralized in Uganda; TB cases requiring hospitalization or advanced clinical care, such as pediatric TB or drug-resistant TB, may be diagnosed at these facilities in the community but are referred to referral hospitals for their treatment and management and would not be included in this analysis. All facilities providing TB treatment in Uganda are expected to follow the national guidelines for TB treatment, although variation in implementation may exist and additional services (such as laboratory tests) may incur additional charges at private facilities. In urban settings, the national guidelines recommend that patients or their treatment supporters report to the treating facility to receive their anti-TB drugs every two weeks during the intensive phase and every four weeks during the continuation phase.

### Data collection

Demographic and clinical data were abstracted retrospectively from the Facility TB Registers, including treatment facility, parish of residence, age at diagnosis, sex, HIV status, site of disease (pulmonary vs. extrapulmonary), treatment regimen, diagnostic test results (sputum microscopy and Xpert MTB/RIF [Cepheid, Inc., Sunnyvale, California, USA]), date of treatment initiation, and treatment outcome. Data were abstracted directly as written in the registers with guidance from health facility staff as needed. Study data were collected and managed using REDCap (Research Electronic Data Capture) [12, 13] hosted at Johns Hopkins Bloomberg School of Public Health.

### Measurement of primary exposure and outcome

We used reported area of residence to calculate two measures of distance from residence to the health facility where the patient chose to receive TB treatment. The TB registers do not capture patient addresses but do have information on the administrative area of residence, the smallest of which is the parish with a median size of 0.13 km^2^ and median population of 23,041 within Kampala. The centroid of the parish of residence was used to estimate each patient’s location of residence based on parish boundaries provided by Uganda Bureau of Statistics 2014 census data. We calculated Euclidean distance as a straight line from parish centroid to each health facility using ArcGIS (ESRI, Redlands, California, USA). Additionally, we used OpenStreetMap (OpenStreetMap Foundation, Cambridge, United Kingdom) to define road networks and calculated travel distance based on the shortest available route using the Network Analysis tool in ArcGIS.

Treatment outcomes following the World Health Organization (WHO) definitions were abstracted from the Facility TB Registers. “Unfavorable” outcomes included treatment failure, death, and loss to follow-up (including those with a documented outcome of default); patients with no documented outcome were not included in the analysis, although we performed a sensitivity analysis in which these patients were considered to have unfavorable outcomes. These were compared to “favorable” treatment outcomes of treatment completion or cure (also called treatment success). Patients with an outcome of “transferred out” (with no additional treatment outcome information from their receiving facility) were excluded.

### Facility TB notification rate

We calculated an average annual “facility TB notification rate” for each parish, which we defined as the annual average number of cases from the parish reported at the six facilities divided by the parish’s 2014 population from the Uganda Bureau of Statistics [14]. We used Poisson regression to assess the association of Euclidean distance from the parish centroid to the facility where the patients received TB treatment on facility TB notification rates.

### Distance to TB treatment facility and treatment outcomes

To measure the association between distance from residence to TB treatment facility and treatment outcome at the individual level, our primary exposure was Euclidean distance categorized into four categories: < 2 km, 2 to < 5 km, 5 to < 10 km, and ≥ 10 km. These categories were chosen based on the following rationale: < 2 km is walking distance and therefore distance should not represent significant barriers to care; 2 to < 5 km is still quite close but may require additional means of transport or additional travel time; 5 to < 10 km is within the urban area but may take significant time and/or resources to travel; ≥10 km represents a significant investment in time and resources to reach the facility. For additional analyses using shortest available route travel distance, we used the same four distance categories. We also considered a binary exposure with distance dichotomized as < 2 km or ≥ 2 km. Our outcome of interest was unfavorable treatment outcome, which was defined as above.

Patient characteristics were compared across the four exposure categories for Euclidean distance using chi-square tests. Risk factors of interest were defined a priori as characteristics known to be associated with TB treatment outcomes and that conceivably could lead to differences in care-seeking behavior and choice of TB treatment facility, and included: age, sex, HIV status, site of disease (pulmonary vs. extrapulmonary), lack of bacteriologic confirmation (positive sputum microscopy or GeneXpert), year of treatment, and treatment facility. We estimated the relative risk as a measure of association between unfavorable treatment outcomes and Euclidean distance, modeling distance both in four categories (with reference to the < 2 km category) and as binary, using simple and multivariable Poisson regression with robust variance. All risk factors of interest were included in the multivariable model regardless of statistical significance. We analyzed associations between travel distance and unfavorable treatment outcomes in similar fashion. In a sub-analysis, we also analyzed death and loss to follow up as separate outcomes compared to favorable treatment outcomes.

## Results

### Study population

**Fig. 1:**
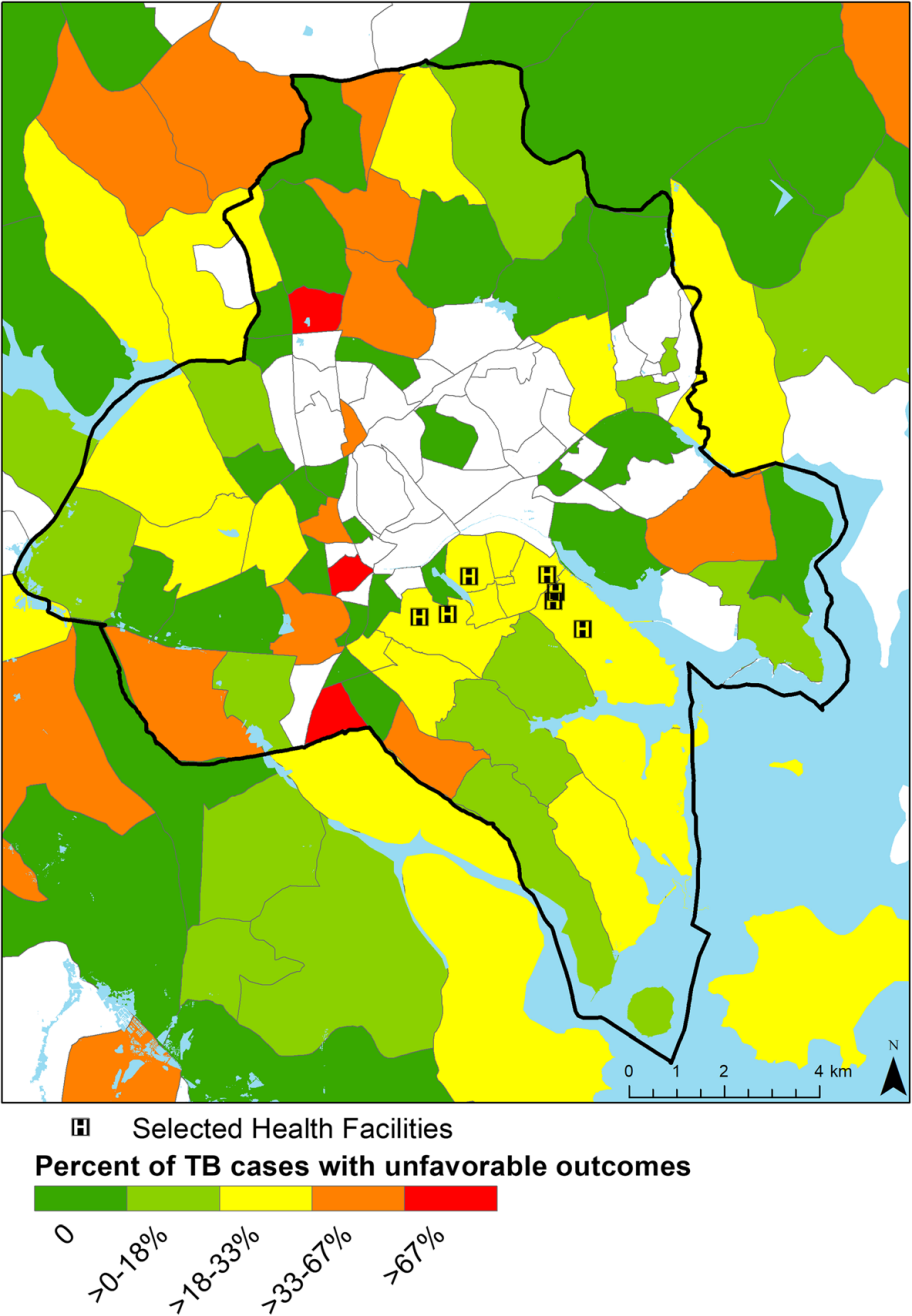
Percentage of TB Cases with unfavorable treatment outcomes by Parish in Kampala^1^ District and surrounding areas, 2014–2016. ^**1**^ Parishes further outside Kampala not displayed. Dark line indicates Kampala District boundary. *Parish boundaries and population provided by Uganda Bureau of Statistics. Maps created using ESRI ArcGIS 10.7.1*

From 2014 to 2016, 2251 patients initiated TB treatment at the six study facilities, of whom 2146 (95.3%) had a documented residential information in the Facility TB register. Patients came from 181 parishes in 28 districts throughout Uganda. We excluded 261 (12.2%) patients whose listed parish of residence information could not be matched to a parish listed in the 2014 Uganda Bureau of Statistics Census and an additional 16 participants from analyses of travel distance who could not be linked due to lack of road network connectivity. We excluded 109 (5.8%) TB patients who were transferred out as we were unable to determine their final treatment outcome. Among 1691 patients with reported outcomes, favorable treatment outcomes were seen in 1352 (80.0%) of TB patients; 85 (4.8%) TB patients had no documented treatment outcomes. Figure 1 shows the proportion of TB cases with unfavorable treatment outcomes by parish for Kampala District and surrounding areas.

### Facility TB notification rate

**Fig. 2:**
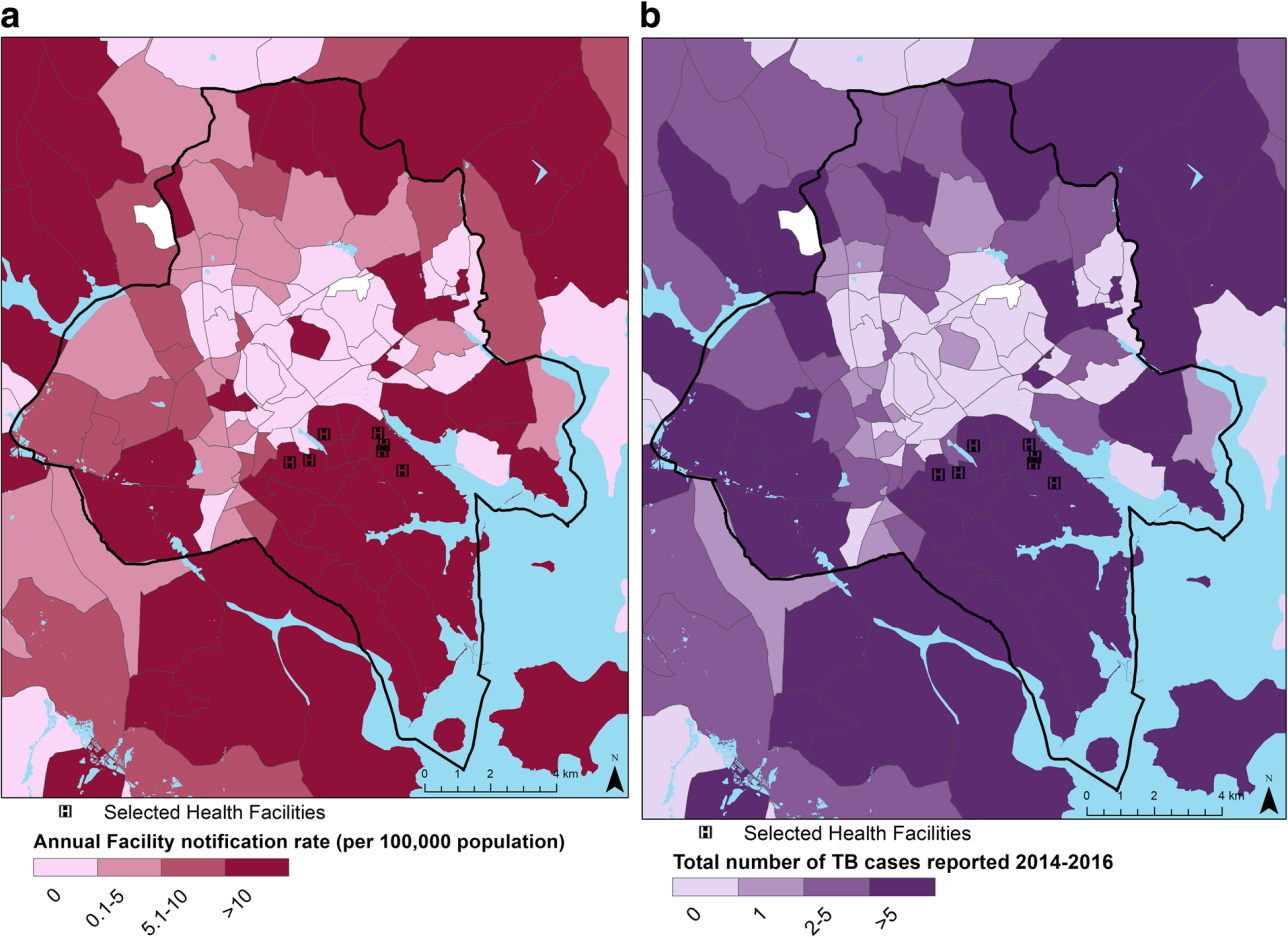
TB Facility Notification by Parish in Kampala District^1^ and surrounding areas for six Facilities serving Kisugu and Wabigalo Parishes^2^, 2014–2016 **a**. Annual Facility Notification Rate per 100,000 population (based on 2014 census). **b**. Total Count of Facility Notified TB Cases. ^**1**^ Parishes further outside Kampala not displayed. Dark line indicates Kampala District boundary. ^**2**^ Facility Notification Rates and Total Number of TB Cases Reported are from six study facilities providing TB treatment and do not represent all TB cases diagnosed at all facilities within each parish. *Parish boundaries and population provided by Uganda Bureau of Statistics. Maps created using ESRI ArcGIS 10.7.1*

Average annual parish-level facility TB notification rates ranged from 0 to 327 TB cases per 100,000 population (Fig. 2). Facility notification rates decreased by 4% with each additional kilometer from the parish centroid to the health facility where the patient received TB treatment (rate ratio 0.96 [95% CI 0.95, 0.97]).

### Distance to TB treatment facility and treatment outcomes

The median Euclidean distance from the centroid of parish of residence to health facility where the patient chose to receive TB treatment was 3.7 km. While many patients lived < 2 km from their chosen facility (34%), nearly half of patients lived 2–10 km from their facility (49%), and 17% lived ≥10 km from their facility. Across the four distance exposure groups, there were differences in age, sex, HIV status, disease site, laboratory confirmation of disease, year of treatment initiation, and health facility (all *p* < 0.05) (Table 1). People living ≥2 km from the facility were more likely to be female (42% vs 34%), HIV positive (53% vs 46%), have extrapulmonary disease (17% vs. 8%), and lack bacteriologic confirmation of disease (37% vs. 24%) compared to those living < 2 km from the facility.

**Table 1:** Patient characteristics by distance from residence to TB health facility

	Total	Euclidean Distance Categories				***p***-value
		< 2 km	2 to < 5 km	5 to < 10 km	≥ 10 km	
	n (%)	n (%)	n (%)	n (%)	n (%)	
**Total**	1776 (100)	606 (34)	459 (26)	407 (23)	304 (17)	
**Age (years) (*N*** **= 1774)**^**a**^						< 0.001
0–14	87 (5)	21 (3)	20 (4)	26 (6)	20 (7)	
15–24	366 (21)	142 (23)	95 (21)	81 (20)	48 (16)	
25–34	627 (35)	224 (37)	177 (39)	140 (35)	86 (29)	
35–44	398 (22)	122 (20)	113 (25)	93 (23)	70 (23)	
45–54	200 (11)	66 (11)	37 (8)	48 (12)	49 (16)	
55–64	61 (3)	21 (3)	12 (3)	11 (3)	17 (6)	
≥ 65	35 (2)	10 (2)	4 (1)	7 (2)	14 (5)	
**Male (*N*** **= 1773)**^**a**^	1079 (61)	401 (66)	286 (62)	219 (54)	173 (57)	0.001
**HIV Positive (*N*** **= 1770)**^**a**^	887 (50)	275 (45)	263 (57)	202 (50)	147 (49)	0.002
**Pulmonary TB (*N*** **= 1765)**	1513 (86)	552 (92)	395 (86)	323 (80)	242 (80)	< 0.001
**Lack of bacteriologic confirmation (*N*** **= 1776)**	569 (32)	142 (23)	132 (29)	168 (41)	127 (42)	< 0.001
**Year of treatment initiation (N = 1773)**^**a**^						0.289
2014	622 (35)	200 (33)	171 (37)	149 (37)	102 (34)	
2015	558 (31)	212 (35)	131 (29)	126 (31)	89 (30)	
2016	593 (33)	194 (32)	156 (34)	132 (32)	111 (37)	
**Facility (N = 1776)**						< 0.001
Kisugu Health Center (public)	441 (24)	284 (47)	84 (18)	51 (13)	22 (7)	
Alive Medical Services	383 (22)	112 (18)	149 (32)	55 (14)	67 (22)	
International Hospital Kampala	178 (10)	92 (15)	27 (6)	37 (9)	22 (7)	
Kibuli Muslim Hospital	184 (10)	49 (8)	28 (6)	63 (15)	44 (14)	
St. Francis Hospital - Nsambya	578 (33)	60 (10)	169 (37)	201 (49)	148 (49)	
Meeting Point	12 (1)	9 (2)	2 (0)	0 (0)	1 (0)	
**Treatment Outcome (N = 1776)**						< 0.001
Cured	919 (51)	342 (56)	239 (52)	194 (48)	144 (47)	
Complete	433 (24)	101 (17)	108 (24)	127 (31)	97 (32)	
Failure	21 (1)	4 (1)	9 (2)	7 (2)	1 (0)	
Died	167 (9)	38 (6)	48 (10)	44 (11)	37 (12)	
Lost to Follow Up (including Default)	151 (9)	88 (15)	29 (6)	22 (5)	12 (4)	
Unknown/Missing	85 (5)	33 (5)	26 (6)	13 (3)	13 (4)	

^a^ Ns below the total of 1776 indicate data missing for that particular variable

In simple and multivariable Poisson regression models, no significant association was seen between the four categories of distance and unfavorable treatment outcomes (Table 2). In analysis of a binary measure of distance, the adjusted relative risk [aRR] for unfavorable treatment outcomes was 0.87 (95% CI 0.70, 1.07) for patients who lived ≥2 km from the facility where they chose to receive TB treatment compared to those living within 2 km. Patients who were HIV positive (aRR 1.72 [95%CI 1.36, 2.17]), over the age of 65 years (aRR 2.53 [95%CI 1.59, 4.04]), or lacked bacteriologic confirmation of TB (aRR 1.57 [95%CI 1.27, 1.94]) were more likely to have unfavorable treatment outcomes. Patients aged less than 14 years (aRR 0.44 [95%CI 0.21, 0.90]) or receiving TB treatment at St. Francis Hospital-Nsambya (aRR 0.60 [95%CI 0.45, 0.80], compared to Kisugu Health Centre) had lower risk of unfavorable treatment outcomes.

**Table 2:** Crude and Adjusted Relative Risks for unfavorable TB treatment outcomes (death, treatment failure, or loss to follow-up) compared to favorable TB treatment outcomes

	Crude RR (95% CI)	Adjusted RR (95% CI)
**Euclidean Distance**		
< 2 km	*Reference*	*Reference*
2 to < 5 km	0.88 (0.69, 1.12)	0.91 (0.70, 1.17)
5 to < 10 km	0.82 (0.63, 1.06)	0.88 (0.68, 1.15)
> 10 km	0.76 (0.56, 1.02)	0.77 (0.57, 1.04)
**Age at diagnosis**		
0–14 years	0.42 (0.20, 0.87)	0.44 (0.21, 0.90)
15–24 years	0.63 (0.46, 0.87)	0.79 (0.57, 1.08)
25–34 years	*Reference*	*Reference*
35–44 years	1.03 (0.80, 1.32)	0.97 (0.75, 1.25)
45–54 years	1.35 (1.02, 1.77)	1.18 (0.89, 1.57)
55–64 years	1.25 (0.79, 1.98)	1.23 (0.79, 1.92)
> 65 years	1.96 (1.25, 3.06)	2.53 (1.59, 4.04)
**Male sex**	1.12 (0.92, 1.36)	1.08 (0.88, 1.32)
**HIV Positive**	1.83 (1.50, 2.24)	1.72 (1.36, 2.17)
**Pulmonary TB**	0.85 (0.66, 1.10)	1.06 (0.80, 1.40)
**Lack of bacteriologic confirmation**	1.47 (1.22, 1.78)	1.57 (1.27, 1.94)
**Treatment Start year**		
2014	*Reference*	*Reference*
2015	0.89 (0.71, 1.12)	0.88 (0.70, 1.11)
2016	0.86 (0.69, 1.09)	0.89 (0.71, 1.11)
**Facility**		
Kisugu Health Center (public)	*Reference*	*Reference*
Alive Medical Services	1.19 (0.93, 1.53)	0.97 (0.74, 1.27)
International Hospital Kampala	0.99 (0.70, 1.40)	0.84 (0.59, 1.20)
Kibuli Muslim Hospital	1.01 (0.72, 1.40)	0.97 (0.70, 1.35)
St. Francis Hospital - Nsambya	0.63 (0.48, 0.83)	0.60 (0.45, 0.80)
Meeting Point	0.76 (0.21, 2.72)	0.55 (0.15, 2.03)

In a sub-analysis evaluating death and loss to follow-up separately, distances of ≥2 km from residence to facility chosen for TB treatment were associated with an increased risk of death but decreased risk of loss to follow up (Table 3). Comparing those living ≥2 km from the facility to those living within 2 km, the adjusted RR for death was 1.42 (95% CI 0.99, 2.03) and the adjusted RR for loss to follow up was 0.57 (95% CI 0.41, 0.78). Risk factors for death included older age (55–64 years or 65+ years), being HIV positive, and lacking bacteriologic confirmation of disease (Table 3). We found no additional risk factors for loss to follow-up during TB treatment.

**Table 3:** Adjusted relative risks for Death and Loss to Follow up during TB treatment compared to favorable TB treatment outcomes

	Death	Lost to Follow Up
	Adjusted RR (95% CI)	Adjusted RR (95% CI)
**Euclidean Distance**		
< 2 km	*Reference*	*Reference*
2 to < 5 km	1.48 (0.99, 2.22)	0.59 (0.38, 0.89)
5 to < 10 km	1.38 (0.90, 2.12)	0.61 (0.39, 0.95)
> 10 km	1.35 (0.86, 2.10)	0.45 (0.25, 0.81)
**Age at diagnosis**		
0–14 years	0.59 (0.24, 1.46)	0.28 (0.07, 1.12)
15–24 years	0.59 (0.30, 1.14)	0.80 (0.53, 1.22)
25–34 years	*Reference*	*Reference*
35–44 years	1.38 (0.94, 2.02)	0.65 (0.42, 0.99)
45–54 years	1.68 (1.11, 2.54)	0.64 (0.36, 1.12)
55–64 years	2.08 (1.23, 3.51)	0.51 (0.16, 1.58)
> 65 years	6.44 (3.69, 11.27)	0.77 (0.20, 3.02)
**Male sex**	0.98 (0.74, 1.31)	1.31 (0.94, 1.83)
**HIV Positive**	3.28 (2.23, 4.81)	1.05 (0.73, 1.51)
**Pulmonary TB**	0.93 (0.66, 1.30)	1.50 (0.78, 2.86)
**Lack of bacteriological confirmation**	2.14 (1.59, 2.89)	1.45 (0.99, 2.13)
**Treatment Start year**		
2014	*Reference*	*Reference*
2015	0.78 (0.55, 1.10)	0.88 (0.61, 1.26)
2016	0.81 (0.59, 1.13)	0.88 (0.61, 1.27)
**Facility**		
Kisugu Health Center (public)	*Reference*	*Reference*
Alive Medical Services	1.02 (0.63, 1.66)	1.10 (0.74, 1.62)
International Hospital Kampala	1.41 (0.83, 2.39)	0.60 (0.34, 1.08)
Kibuli Muslim Hospital	1.48 (0.90, 2.44)	0.83 (0.49, 1.41)
St. Francis Hospital - Nsambya	0.92 (0.59, 1.45)	0.25 (0.14, 0.46)
Meeting Point	1.72 (0.51, 5.77)	*excluded*

Meeting Point reported no patients lost to follow up

### Travel distance

Travel distance using the shortest available route was strongly correlated with Euclidean distance (R^2^ = 0.98) but was on average 19% further than Euclidean distance (95% CI 18, 20%). While 31.1% of participants were reclassified to a different distance category if travel distance was used instead of Euclidean distance, there were no substantive differences in the association between distance from facility chosen for TB treatment and treatment outcomes when using travel distance as the exposure compared to Euclidean distance (Tables in supplement).

## Discussion

This analysis of 1774 patients treated for TB across six urban clinics in the Makindye division of Kampala, Uganda, was suggestive of a protective association between longer distance from home to chosen treatment facility and composite unfavorable treatment outcomes. Facility notification rates for the included treatment facilities were high in parishes nearest to the facilities but were also high for some parishes far from the facilities. Nevertheless, despite the high density of TB treatment facilities in Kampala, 66% of patients starting TB treatment in these six facilities lived more than ≥2 km from the treating facilities (Table 1); compared to those who lived within 2 km of the facility, those living more remotely were 42% more likely to die but 43% less likely to be lost to follow-up.

Most patients in our study setting live within 2 km of multiple facilities and therefore can choose where they want to receive TB care. Patients may choose to seek care at a facility further away due to stigma against TB and a desire to hide their TB status [15–17], convenience due to work or other travel [18], or perception of better care, particularly at private facilities [18]. This dynamic may explain the differences seen when considering death versus loss to follow up as an outcome. Death during TB treatment in this setting (where multidrug resistance is uncommon) likely reflects a patient’s severity of illness when diagnosed, whereas loss to follow-up may more closely reflect patient motivation and health system investment. Thus, patients who choose to travel more than 2 km to be treated may be those who experience other barriers to seeking care (e.g., stigma, job-related time limitations, unease with the healthcare system) which can cause delays in diagnosis and treatment. Such delays are associated with increased disease severity [19] and higher corresponding mortality rates [20–22]. However, once treatment is initiated, these patients who are sicker and willing to travel longer distances may be more motivated to adhere to treatment, thereby reducing losses to follow-up. These findings illustrate how important distinctions between these two outcomes may be obscured when considering unfavorable treatment outcome as a composite measure.

Our study fills an important gap in knowledge regarding barriers to care and TB treatment outcomes. Prior studies have had mixed results regarding the effect of distance on delays in TB diagnosis and initiation of TB treatment [2–4]. Our study suggests that, once treatment is initiated, distance is not associated with overall unfavorable treatment outcomes. Other studies have highlighted economic, socio-cultural, and health system barriers to care in high-burden settings as access-related risk factors for poor treatment outcomes [23]; the current research suggests that geographic distance to treatment facility may not be a strong measure of access to care, particularly in the urban sub-Saharan African setting. Additionally, our finding that travel distance using the shortest available route does not change our results compared to using Euclidean distance is in contrast with other research [24]. This may reflect the high population density and informal road network in our setting, such that travel paths are generally direct and the differences between Euclidean and travel distances are small, particularly if walking or using boda bodas (motorbike taxis) for transport.

This analysis does have some key limitations, largely due to the limited data available in facility TB registers. For example, these registers do not contain data on precise address of residence, thus limiting our measurement of distance to that of the parish centroid. Nevertheless, on average, participants will live within 500 m of the parish centroid, making major bias at the scale of our distance categories less likely. Additionally, while Euclidean distance does not directly capture the distance traveled to seek care, our assessment of travel distance yielded comparable results. Due to the limited data available in the TB registers, we are unable to assess the contribution of broader barriers to health care access, including economic, socio-cultural, and health system barriers. These factors may overlap or interact with geographic barriers. Additionally, we could not assess patients’ reasons for choosing particular facilities; further qualitative research could help to elucidate these motivations. Our study includes patients attending facilities in a densely crowded urban area, and our findings may not generalize to other settings (e.g., rural areas) where facilities are further apart and patients have fewer treatment options. While we do not have complete capture of any geographic region (e.g., people living in Kisugu and Wabigalo parishes) due to our sampling frame based on health facilities, we do have full capture of every patient seeking care at these facilities. Our final models excluded more than 20% of TB cases seen at these facilities due to missing data on either residence or treatment outcome; while our sensitivity analysis showed no major effect of excluding those missing treatment outcomes, we may have selection bias if those included in our analysis are not representative of those missing residential information in regards to the association of distance on treatment outcomes. Finally, since we only considered patients enrolled in care, we could not assess the role of geographic barriers in limiting initial access to care.

## Conclusion

Distance from home residence to TB treatment facility was not associated with overall unfavorable treatment outcomes in this urban Ugandan population, but was associated with increased risk of death and decreased risk of loss to follow up. These findings suggest that those who seek care further from home may do so at a more advanced disease state, but once enrolled they may be more likely to remain in treatment. This is important for TB control programs to consider, as they may need to invest in programs that decrease delays in diagnosis among those living further away and improving treatment adherence among those who live closer to facilities. A detailed understanding of the patient population and the varying experiences of that population is key to appropriately focusing resources to improve TB treatment outcomes.

## Supplementary information

**Additional file 1.** Supplemental Results.

## Data Availability

The dataset used for this analysis is available on the Johns Hopkins University Data Archive (https://doi.org/10.7281/T1/LW2H2F).

## Abbreviations

aRR: Adjusted relative risk
CI: Confidence interval
ESRI: Environmental Systems Research Institute
HIV: Human immunodeficiency virus
REDCap: Research electronic data capture
TB: Tuberculosis
WHO: World Health Organization
