# Supplementary material for: Is distance associated with tuberculosis treatment outcomes? A retrospective cohort study in Kampala, Uganda
Source: BMC Infect Dis. 2020 Jun 11;20:406. doi: 10.1186/s12879-020-05099-z (PMC7291553; doi:10.1186/s12879-020-05099-z)
Supplement: Supplementary file 1 — Additional file 1. Supplemental Results. [file 12879_2020_5099_MOESM1_ESM.docx]

**Supplemental Material**

Adjusted relative risks for unfavorable TB treatment outcomes using Euclidean and travel distance

|  | **Euclidean Distance** | **Travel Distance** |
| --- | --- | --- |
|  | **Adjusted RR (95% CI)** | **Adjusted RR (95% CI)** |
| **Distance** |  |  |
| <2 km | *Reference* | *Reference* |
| 2 to <5 km | 0.91 (0.70, 1.17) | 0.94 (0.70, 1.26) |
| 5 to <10 km | 0.88 (0.68, 1.15) | 0.96 (0.71, 1.31) |
| >10 km | 0.77 (0.57, 1.04) | 0.79 (0.56, 1.10) |
| **Age at diagnosis** |  |  |
| 0-14 years | 0.44 (0.21, 0.90) | 0.44 (0.21, 0.91) |
| 15-24 years | 0.79 (0.57, 1.08) | 0.79 (0.57, 1.09) |
| 25-34 years | *Reference* | *Reference* |
| 35-44 years | 0.97 (0.75, 1.25) | 0.97 (0.75, 1.25) |
| 45-54 years | 1.18 (0.89, 1.57) | 1.19 (0.90, 1.59) |
| 55-64 years | 1.23 (0.79, 1.92) | 1.25 (0.80, 1.95) |
| >65 years | 2.53 (1.59, 4.04) | 2.54 (1.59, 4.04) |
| **Male** | 1.08 (0.88, 1.32) | 1.09 (0.89, 1.33) |
| **HIV Positive** | 1.72 (1.36, 2.17) | 1.71 (1.35, 2.16) |
| **Pulmonary TB** | 1.06 (0.80, 1.40) | 1.05 (0.79, 1.39) |
| **Lack of bacteriological confirmation** | 1.57 (1.27, 1.94) | 1.58 (1.28, 1.95) |
| **Treatment Start year** |  |  |
| 2014 | *Reference* | *Reference* |
| 2015 | 0.88 (0.70, 1.11) | 0.89 (0.71, 1.12) |
| 2016 | 0.89 (0.71, 1.11) | 0.89 (0.71, 1.12) |
| **Facility** |  |  |
| Kisugu Health Center (public) | *Reference* | *Reference* |
| Alive Medical Services | 0.97 (0.74, 1.27) | 0.94 (0.72, 1.23) |
| International Hospital Kampala | 0.84 (0.59, 1.20) | 0.84 (0.59, 1.20) |
| Kibuli Muslim Hospital | 0.97 (0.70, 1.35) | 0.93 (0.67, 1.30) |
| St. Francis Hospital - Nsambya | 0.60 (0.45, 0.80) | 0.57 (0.43, 0.75) |
| Meeting Point | 0.55 (0.15, 2.03) | 0.54 (0.14, 2.05) |

Sensitivity Analysis comparing adjusted relative risks for unfavorable TB treatment outcomes including and excluding patients with no documented outcomes

|  | **Unfavorable Outcomes** | **Unfavorable + Unknown Outcomes** |
| --- | --- | --- |
|  | **Adjusted RR (95% CI)** | **Adjusted RR (95% CI)** |
| **Euclidean Distance** |  |  |
| <2 km | *Reference* | *Reference* |
| 2 to<5 km | 0.91 (0.70, 1.17) | 0.94 (0.78, 1.14) |
| 5 to <10 km | 0.88 (0.68, 1.15) | 0.90 (0.73, 1.10) |
| >10 km | 0.77 (0.57, 1.04) | 0.90 (0.72, 1.12) |
| **Age at diagnosis** |  |  |
| 0-14 years | 0.44 (0.21, 0.90) | 0.69 (0.45, 1.05) |
| 15-24 years | 0.79 (0.57, 1.08) | 0.90 (0.72, 1.13) |
| 25-34 years | *Reference* | *Reference* |
| 35-44 years | 0.97 (0.75, 1.25) | 0.96 (0.80, 1.17) |
| 45-54 years | 1.18 (0.89, 1.57) | 1.06 (0.85, 1.33) |
| 55-64 years | 1.23 (0.79, 1.92) | 1.11 (0.78, 1.59) |
| >65 years | 2.53 (1.59, 4.04) | 1.93 (1.31, 2.83) |
| **Male** | 1.08 (0.88, 1.32) | 1.09 (0.94, 1.26) |
| **HIV Positive** | 1.72 (1.36, 2.17) | 1.40 (1.18, 1.66) |
| **Pulmonary TB** | 1.06 (0.80, 1.40) | 1.01 (0.82, 1.24) |
| **Lack of bacteriological confirmation** | 1.57 (1.27, 1.94) | 1.56 (1.33, 1.84) |
| **Treatment Start year** |  |  |
| 2014 | Reference | Reference |
| 2015 | 0.88 (0.70, 1.11) | 0.94 (0.78, 1.12) |
| 2016 | 0.89 (0.71, 1.11) | 1.04 (0.88, 1.23) |
| **Facility** |  |  |
| Kisugu Health Center | Reference | Reference |
| Alive Medical Services | 0.97 (0.74, 1.27) | 1.09 (0.89, 1.33) |
| International Hospital | 0.84 (0.59, 1.20) | 1.15 (0.91, 1.45) |
| Kibuli Muslim Hospital | 0.97 (0.70, 1.35) | 0.96 (0.75, 1.24) |
| St. Francis Hospital - Nsambya | 0.60 (0.45, 0.80) | 0.51 (0.40, 0.65) |
| Meeting Point | 0.55 (0.15, 2.03) | 0.45 (0.12, 1.61) |

Adjusted relative risks for unfavorable TB treatment outcomes by TB Treatment Facility

|  | **Kisugu Health Center** | **Alive Medical Services** | **International Hospital Kampala** | **Kibuli Muslim Hospital** | **St. Francis Hospital - Nsambya** |
| --- | --- | --- | --- | --- | --- |
|  | **Adjusted RR (95% CI)** | **Adjusted RR (95% CI)** | **Adjusted RR (95% CI)** | **Adjusted RR (95% CI)** | **Adjusted RR (95% CI)** |
| **Euclidean Distance** |  |  |  |  |  |
| <2 km | *Reference* | *Reference* | *Reference* | *Reference* | *Reference* |
| ≥2 km | 0.69 (0.47, 1.02) | 0.95 (0.65, 1.38) | 1.00 (0.56, 1.8) | 1.21 (0.63, 2.31) | 0.86 (0.47, 1.60) |
| **Age at diagnosis** |  |  |  |  |  |
| 0-14 years | 0 (0, 0) | 0.24 (0.04, 1.62) | 0.54 (0.13, 2.16) | 1.36 (0.27, 6.82) | 0.51 (0.16, 1.58) |
| 15-24 years | 0.70 (0.40, 1.21) | 1.09 (0.63, 1.88) | 0.91 (0.29, 2.85) | 0.54 (0.16, 1.81) | 0.68 (0.30, 1.51) |
| 25-34 years | *Reference* | *Reference* | *Reference* | *Reference* | *Reference* |
| 35-44 years | 0.89 (0.56, 1.42) | 0.89 (0.56, 1.41) | 0.78 (0.34, 1.78) | 1.24 (0.60, 2.56) | 1.03 (0.59, 1.78) |
| 45-54 years | 0.83 (0.38, 1.80) | 1.17 (0.73, 1.86) | 1.06 (0.47, 2.39) | 0.87 (0.42, 1.80) | 1.56 (0.86, 2.85) |
| 55-64 years | 1.24 (0.48, 3.19) | 0 (0, 0) | 0 (0, 0) | 1.33 (0.67, 2.66) | 2 (0.94, 4.25) |
| >65 years | 2.16 (0.96, 4.87) | 0 (0, 0) | 4.07 (0.62, 26.54) | 0.63 (0.08, 5.35) | 6.16 (2.99, 12.71) |
| **Male** | 1.41 (0.92, 2.17) | 0.89 (0.61, 1.29) | 0.88 (0.48, 1.62) | 1.07 (0.61, 1.85) | 1.15 (0.76, 1.74) |
| **HIV Positive** | 1.44 (0.96, 2.16) | 1.12 (0.66, 1.90) | 1.92 (0.87, 4.27) | 2.07 (1.14, 3.76) | 2.43 (1.55, 3.81) |
| **Pulmonary TB** | 2.41 (0.36, 16.22) | 0.86 (0.50, 1.50) | 2.33 (0.92, 5.95) | 1.03 (0.60, 1.77) | 0.82 (0.49, 1.38) |
| **No bacteriological confirmation** | 1.28 (0.76, 2.15) | 1.2 (0.78, 1.83) | 2.37 (1.12, 4.99) | 4.04 (2.05, 7.97) | 1.36 (0.82, 2.24) |
| **Treatment Start year** |  |  |  |  |  |
| 2014 | *Reference* | *Reference* | *Reference* | *Reference* | *Reference* |
| 2015 | 1.01 (0.65, 1.57) | 0.79 (0.49, 1.26) | 0.75 (0.38, 1.49) | 0.54 (0.27, 1.07) | 1.22 (0.75, 1.96) |
| 2016 | 1.11 (0.71, 1.73) | 1.08 (0.72, 1.61) | 0.68 (0.23, 2.00) | 0.63 (0.36, 1.10) | 0.7 (0.42, 1.16) |
